# Potential of Alkali–Silica Reactivity of Unexplored Local Aggregates as per ASTM C1260

**DOI:** 10.3390/ma15196627

**Published:** 2022-09-24

**Authors:** Safeer Abbas, Iqtidar Hussain, Fahid Aslam, Ali Ahmed, Syed Asad Ali Gillani, Aqsa Shabbir, Ahmed Farouk Deifalla

**Affiliations:** 1 Civil Engineering Department, University of Engineering and Technology, Lahore 54890, Pakistan; 2 Department of Civil Engineering, College of Engineering in Alkharj, Prince Sattam bin Abdulaziz University, Al-Kharj 11942, Saudi Arabia; 3 Structural Engineering Department, Faculty of Engineering and Technology, Future University in Egypt, New Cairo 11835, Egypt

**Keywords:** ASR, mechanical properties, expansion, Orakzai aggregates, ASTM C1260

## Abstract

Alkali–silica reaction (ASR) is one of the major durability issues that affect the material degradation and structural performance, compromising the service life of concrete structures. Therefore, this study was planned to investigate the potential of ASR for locally available unexplored and vastly used aggregates, as per ASTM C1260. Aggregates from five different sources (Shalozan, Abbotabad, Orakzai, Swabi and Sada) were procured from their respective crusher sites. Mineralogical components of these aggregates were studied using the petrographic analysis. Cube, prism and mortar bar specimens were cast using mixture design in accordance with ASTM C1260 and placed in sodium hydroxide solution at 80 °C for 90 days. Identical specimens were also cured in water for the purpose of comparison. It was observed that mortar bar expansion of Orakzai aggregate was higher among the other tested aggregates and greater than 0.20% at 28 days, indicating the reactive nature according to ASTM C1260. Petrographic analysis also revealed the presence of reactive silica (quartzite) in the tested Orakzai source. It was observed that the compressive and flexural strengths of specimens exposed to ASR conducive environment was lower than the identical specimens placed in water. For instance, an approximately 9% decrease in compressive strength was observed for Orakzai aggregates exposed to ASR environment at 90 days compared to similar specimens placed in water curing. Moreover, microstructural analysis showed the development of micro-cracks for specimens incorporating Orakzai source aggregates. This study assists the construction stakeholders for the potential of unexplored local aggregates with regard to ASR before its utilization in mega construction projects.

## 1. Introduction

Alkali–silica reaction (ASR) in concrete is a chemical reaction that occurs when hydroxyl ions in concrete pore fluid combine with aggregate reactive silica [1]. Alkali–silica gel forms as a result of this reaction. ASR gel itself is very stable and does not induce cracking or other damaging effects; however, when in contact with water, it can swell [2]. Due to the swelling of this gel, the concrete begins to expand, causing stresses in the surrounding concrete, which results in internal microcracks or fractures [3]. The following chemical reactions (Equations (1)–(3)) summarize the three-step process of the alkali–silica reaction mechanism [1]. Initially, the siloxane bonds (Si-O-Si) are disintegrated by the hydroxyl ions (OH^−^) and conversion of formed weak silicic acid into alkali silicate (alkali–silica gel) takes place (Equations (1) and (2)). Afterwards, water absorption will lead to the subsequent expansion of the alkali–silicate gel (Equation (3)).

(1) $$\mathrm{Si}-O-\mathrm{Si}+R^{+}+{\mathrm{OH}}^{-}\to \underset{\;\mathrm{Alkali}\;\mathrm{silicate}}{\mathrm{Si}-O-R}+\underset{\mathrm{silicic}\;\mathrm{acid}}{H-O-\mathrm{Si}}$$

(2) $$R^{+}+{\mathrm{OH}}^{-}+\underset{\;\mathrm{Silicic}\;\mathrm{acid}}{H-O-\mathrm{Si}}\to \underset{\mathrm{Alkali}\;\mathrm{silicate}}{\mathrm{Si}-O-R}+H_{2}O$$

(3) $$\underset{\mathrm{Alkali}\;\mathrm{silicate}}{\mathrm{Si}-O-R}+{\mathrm{nH}}_{2}O\to \mathrm{Si}-O^{-}-{\left(H_{2}O\right)}_{n}+R^{+}$$

It is evident that three key components must interact simultaneously for the reaction to occur on a continuous basis: reactive particles in aggregate (including opal, quartzite, and chert), an adequate amount of alkalis in cement and a source of moisture. A lack of any one of these elements is enough to stop the reaction from happening.

Throughout the last few decades, countless studies on the alkali–silica reaction (ASR) have been conducted. Stanton was the first to identify and study ASR in concrete [4]. When it comes to the physical effects of ASR on concrete, it produces cracking, expansion and changes in mechanical properties. All of these impacts, in turn, lead to the deterioration of concrete structures’ serviceability, durability and load-bearing capability [5]. The development and progression of microcracking is widely thought to be the cause of material property changes [6]. The impact of ASR on the mechanical properties of concrete have been extensively studied through experimental, numerical and analytical investigations due to the importance of concrete mechanical properties in structural integrity, serviceability and capacity evaluations. For example, Munir et al. [7] reported compressive strength loss of 22% for specimens with Mach Hills aggregates under ASTM C1260 [8] exposure conditions, whereas Tuguwali aggregate reported 16% loss under the same circumstances, and overall loss in flexure strength of the samples tested under ASTM C 1260 [8] by Munir et al. [7] was 22 to 34%. Marzouk et al. [9] reported overall reduction of 24% in the compressive strength of normal-strength concrete at 84 days for the specimens exposed to NaOH solution, and reported loss of flexural strength of up to 24% associated with moderately reactive aggregates. Ghafoori et al. [10] has reported reduction in compressive strength of concrete cylinders due to ASR for specimens incorporating reactive aggregates. The findings of the tests revealed that compressive strength was not sensitive to ASR at an early age, but it was considerably damaged at a later age when excessive expansions and cracks occurred. Okpin et al. [11] used various testing standards to study the degradation of mechanical characteristics of concretes casted with three types of aggregate and concluded that compressive strength varied over the testing periods. The strength of concrete gradually increased up to 28 days and then decreased.

Expansion of mortar bars is widely used to access the degradation of concrete due to ASR. Various standard procedures around the globe have been used to measure the ASR expansion, indicating the aggregate’s reactivity. The accelerated mortar bars test is most frequently used around the world. Oberholster and Davis [12] introduced the accelerated mortar bar test in 1986, and it has since been extensively accepted as an accelerated test method for assessing the ASR of aggregate. A strongly alkaline solution is used to submerge the mortar bars (1N NaOH) for a minimum of two weeks kept at 80 °C (ASTM C1260 [8]). ASTM C227 [13] is another method that has been a widely used approach for detecting ASR potential for a long time. This standard, on the other hand, takes nearly six months to complete the test [7]. Furthermore, the exposure parameters in ASTM C227 [13] are not severe enough to produce ASR in a short period of time. Therefore, in the present study, ASTM C1260 [8] was used to access the unexplored locally used aggregates with regard to ASR.

Various previous studies have been conducted in the past on the local aggregates to evaluate their physical and engineering properties. Table 1 summarized the previous studies conducted on local aggregates. Very scant literature is available on the potential of ASR of local aggregates as per ASTM C1260 [8]. Therefore, this study was planned to explore the behavior of local aggregates with regard to ASR. Aggregates specimens were procured from five quarries in the Northern region of Pakistan. These quarries were selected based on their expected use in mega construction projects such as dams. It should be noted that the mechanical properties of concrete structures are significantly influenced by the ASR. Therefore, the most efficient way to avoid concrete deterioration in mega-construction is the initial scrutiny of unexplored aggregates with regard to ASR. Initially, petrographic examination has been conducted on the tested aggregates to explore their minerals and reactive constituents. Cube, prism and mortar bar specimens were cast and exposed to ASR solution as per ASTM C1260 [8]. Identical specimens were also cast and cured under normal water for the purpose of comparison. The extent of concrete damage incorporating tested unexplored aggregates in terms of ASR expansion and loss in mechanical properties under ASR exposure condition in accordance with ASTM C1260 is the main contribution and novelty of this research investigation. This study not only evaluated the potential of unexplored aggregates against ASR, but also helped the stakeholders to use the local aggregates in mega projects with confidence and technical assistance. Moreover, this research made an effort to explore and increase the potential sources of local aggregates suitable for construction without adverse effects on mechanical properties.

## 2. Materials and Specimen Preparation

**Figure 1:**
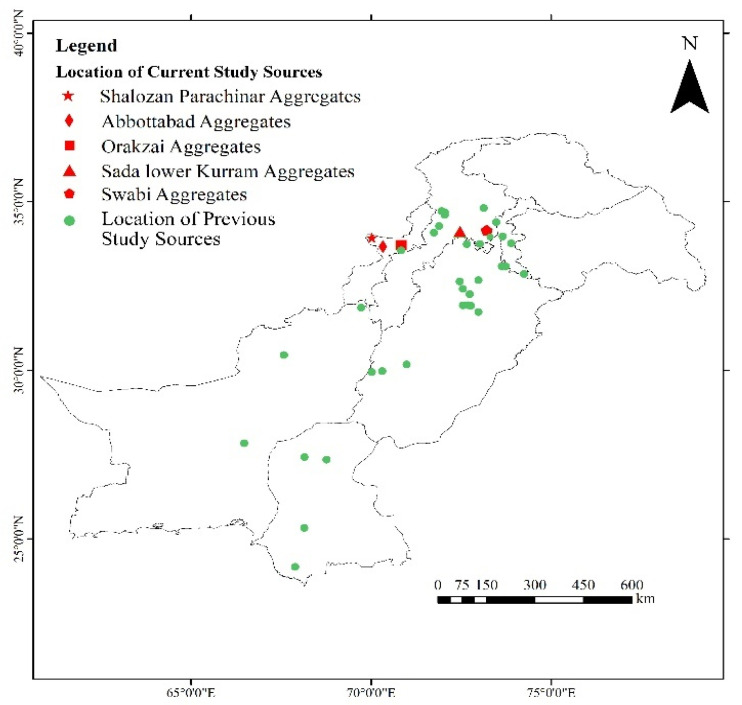
Location of current study aggregates in comparison with aggregates reported in previous studies.

In this study, five different sources of aggregates were evaluated to access the alkali–silica reactivity. The selected sources were Shalozan, Abbottabad, Orakzai, Swabi and Sada aggregates (Table 2 and Table 3). These sources represented the most frequently used aggregates nowadays for mega projects in the local construction industry. Figure 1 shows the location of current study aggregates in comparison with aggregates tested in previous studies. The selected aggregate sources (Shalozan, Abbottabad, Orakzai, Swabi and Sada aggregates) were prepared using blasting of the various hills located in respective regions. After blasting, crushing was performed using a dry process to break huge rock pieces into smaller-sized aggregates. After procuring aggregates from their respective sites, further gradation of aggregates was conducted in accordance with ASTM C1260 [8]. Ordinary Portland cement was used. The mixture was prepared using tap water.

The mixture was prepared using one part cement and 2.25 parts graded aggregates, while maintaining a water-to-cement ratio of 0.47 as per ASTM C1260 [8]. Using an electric mortar mixer, mortar was thoroughly mixed, and specimens were cast. Mortar bars (25 mm × 25 mm × 285 mm), cubes (50 mm × 50 mm × 50 mm) and prisms (40 mm × 40 mm × 160 mm) were cast in two layers and compacted thoroughly. Five specimens were cast for each tested aggregates source at each testing ages. To prevent moisture loss, a plastic covering was placed over the specimens until they were demolded after 24 h.

## 3. Experimental Procedures

To determine the engineering properties of the used cement, different tests were performed, including standard consistency of cement (ASTM C187 [28]), setting time (ASTM C191 [29]), fineness test (passing #200 (ASTM C184 [30]), Blaine air permeability (ASTM C204 [31]) and a soundness test (EN 196-3 [32]). Various tests on the used aggregates were also performed in order to evaluate their physical and chemical properties. These tests included bulk density and void ratio (ASTM C29 [33]), specific gravity and water absorption (ASTM C127 [34]), impact value (BS-812-112 [35]), crushing value (BS-812-110 [36]) and abrasion value (ASTM C535 [37]) tests. Chemical analysis of aggregates was performed on powered specimens in accordance with ASTM C114 [38]. Furthermore, petrographic analysis was conducted as per ASTM C295 [39]. Furthermore, for petrographic analysis, samples were firstly washed and analyzed to identify rock types (petrographic modal analysis) under low and high magnification. Thin sections were also prepared and examined under a polarizing microscope for petrographic analysis and identification of deleterious constituents. The petrographic microscope used was Olympus BX41TF with digital camera DP12.

**Figure 2:**
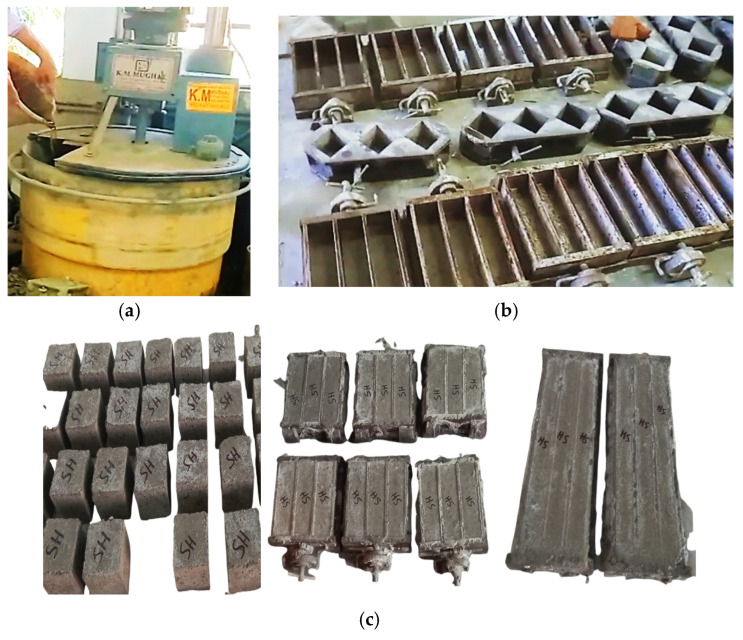

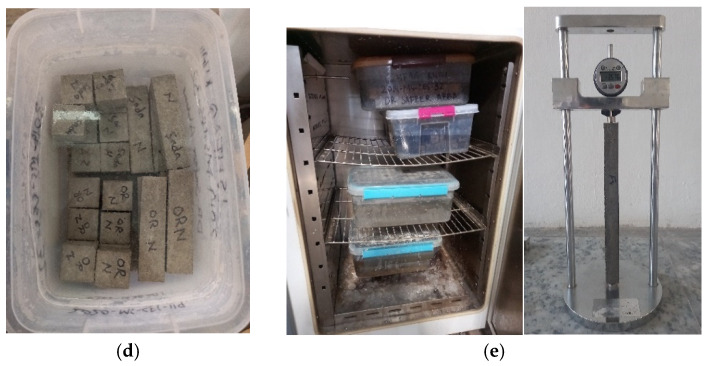
Specimen preparation and testing (**a**) Electrical mixer; (**b**) molds placed on vibratory table; (**c**) casted specimens; (**d**) specimens placed in ASR solution; (**e**) specimens placed in oven and testing using digital length comparator.

Expansion was monitored on mortar bar specimens at 3, 7, 14, 28, 56 and 90 days. Before taking the readings of expansions on the mortar bars, each time digital length comparator reading was adjusted using a calibrated rod. According to ASTM C109 [40], compression strength testing on cubes was performed with the specified loading rate of 1000 N/s, and according to ASTM C348 [41], the flexural strength testing on prisms was performed with the loading rate of 2640 N/s. Testing ages were also 3, 7, 14, 28, 56, and 90 days after ASR exposure to 80 °C. Identical specimens were also cast and placed in water curing and tested for comparison with ASR exposure conditions. Figure 2 shows the specimen preparation and testing procedures.

After demolding of mortar bar expansion specimens, an initial reading of their length was taken using a length comparator in accordance with ASTM C490 [42]. Specimens were immersed in water at 80 °C for 24 h, and again, length change was measured using a length comparator. Afterwards, mortar bar specimens were shifted to NaOH solution at 80 °C. Length change was measured at desired ages. Length change was expressed in percentages and calculated as follows (Equation (4)).

(4) $$L=\frac{L_{x}-L_{i}}{G}\times 100$$

where *L* is the expansion at desired age; *L_x_* is the length comparator reading at desired age; *L_i_* is the initial reading and *G* is the gauge length.

Field emission scanning electron microscope (FESEM) analysis was also conducted to examine the microcracking and associated damages in the tested specimens. The used FESEM was Sigma 500VP Carl Zeiss, Jena, Germany. Small fragments of 3 to 5 mm from the specimens incorporating various aggregates were used for FESEM. Before placing the selected fragments in specimen holder of FESEM, fragments were gold coated using sputter-coating equipment. Specimen fragments were analyzed under various magnifications.

## 4. Results and Discussion

### 4.1. Binder and Aggregates Characteristics

Table 4 shows the results of the physical properties of cement. The results revealed that all physical properties of the cement were within the limits specified by ASTM and European (EN) standards. For instance, the fineness and surface area of cement was more than 90% and 2250 cm^2^/gm, respectively, as specified by ASTM standards. As per ASTM C 151 [43], the autoclave expansion of cement was also well below 0.8%.

Table 5 shows the physical properties of aggregates. The results revealed that each aggregates sources exhibited unique trend of characteristics. The bulk density of all the aggregate sources was in the specified range. Among all the aggregate sources, the bulk density of Swabi aggregates was higher (1500 kg/m^3^), whereas the Sada source had a lower value (1410 Kg/m^3^). Swabi aggregate showed a higher value of specific gravity (2.81) compared to all other tested aggregates sources. However, Shalozan source had the lowest value (2.62). Sada aggregates, on the other hand, had the minimum water absorption (0.22%) compared to Shalozan aggregates (0.58%), which had the maximum absorption of all the tested samples. As per BS-812-112 [35], aggregates are considered strong if the impact values are less than 10, while those with impact values greater than 35 are typically considered weak aggregates for construction. Similarly, aggregate which has crushing values of less than 30% was permissible (BS-812-110 [36]). In this study, the maximum resistance against crushing, abrasion and impact was shown by Swabi aggregates, and the minimum impact value resistance was observed for Abbottabad aggregate. However, minimum resistance against crushing and abrasion was observed for Sada aggregates.

Table 6 shows the results of chemical analysis of aggregates. Results show that presence of all chemical compounds was in accordance with the limits mentioned in ASTM C114 [38]. Among all the tested aggregate sources, the Orakzai aggregates and Shalozan aggregates have higher values of silica that were around 64.5% and 37%, respectively, whereas the Abbottabad source has a lower value of silica that was around 1.55%. It can be argued that the higher values of silica contents in tested Orakzai and Shalozan aggregates will lead towards higher ASR expansion. However, ASR expansion and its damaging effects are dependent on the type, size, amount and reactivity of silica present in aggregates [44,45]. The crystalline silica consists of silicon tetrahedron and oxygen ions. These components are arranged in such a way that maintains the electrical neutrality behavior, leading to formation of a more stable structure [46]. On the other hand, amorphous silica having porous and unstable structure leading to prone ASR [46]. Moreover, it should be noted that the amount of reactive silica present in the aggregates may be very small and cannot be evaluated through chemical analysis. Additionally, the mineralogical composition of aggregates will not exactly determine the extent of the damage due to reactive aggregates. Therefore, it is highly recommended to conduct an experimental laboratory study as per the relevant standard for a complete understanding of the behavior of aggregates with regard to ASR.

All of the tested aggregates have higher values of loss on ignition. For instance, Swabi aggregates showed loss on ignition of around 44%. All other ingredients of the aggregates were within a specified limit of ASTM C114 [38].

### 4.2. Petrographic Examination of Aggregate Samples

#### 4.2.1. Shalozan Aggregate

**Figure 3:**
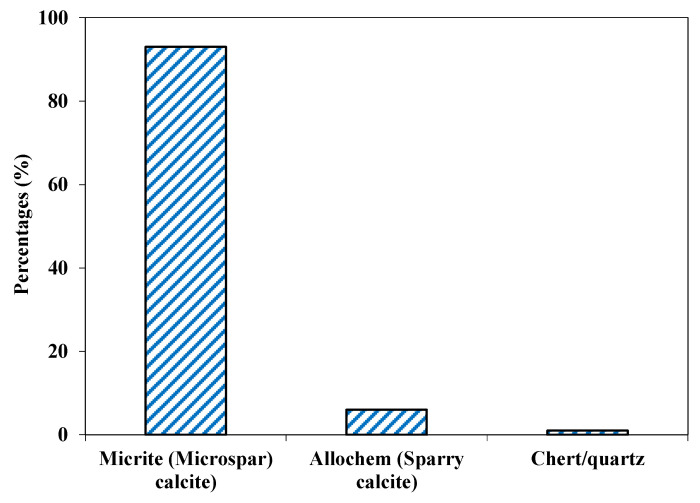
Mineralogical composition of Shalozan aggregates.

**Figure 4:**
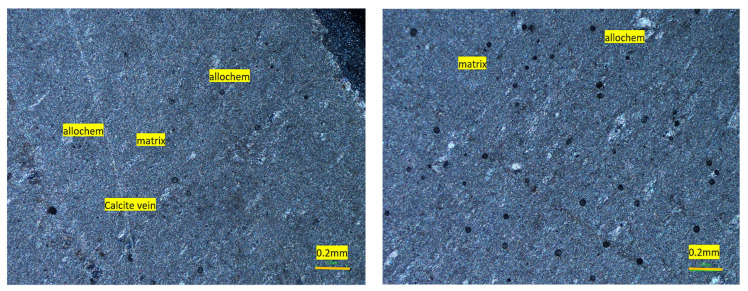
Photomicrographs of mudstone showing few allochems and lime mud matrix (Shalozan aggregate).

This aggregate was fine to medium grained (<0.1 to 0.5 mm) and showed a gray color. Based on petrographic analysis, these fragments were classified as fine-grained limestone (Calcite-Mudstone as per Dunham classification [47]), where small calcite grains (CaCO_3_) formed more than 90% of the samples by volume (Figure 3). Major components were the sparry/micro-sparry calcite (CaCO_3_) formed by neomorphism of fine matrix. A few calcite veins consisting of subhedral grains were also observed (Figure 4), and these formed about 1% of the sample. Quartz/chert (1%) also occurred as subhedral to anhedral grains, and formed a very minor portion of the sample. This studied samples may be suitable for use as aggregate material both in asphalt and concrete work.

#### 4.2.2. Abbottabad Aggregate

**Figure 5:**
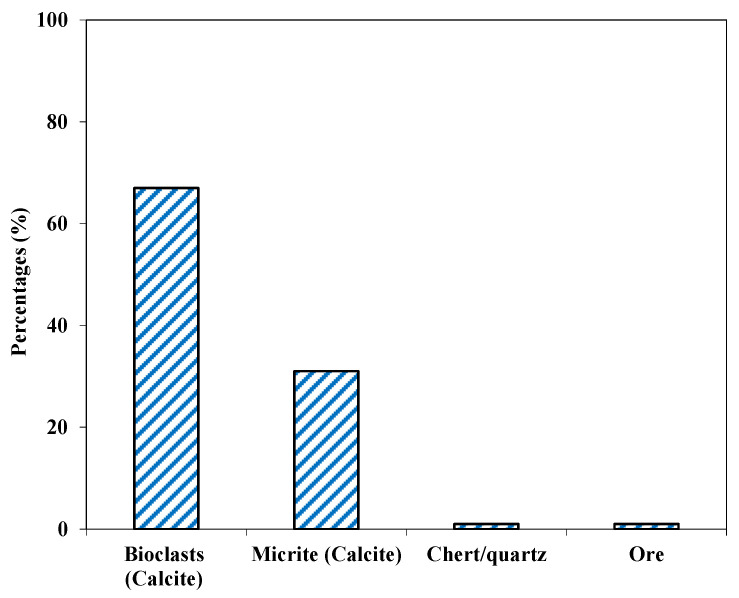
Mineralogical composition of Abbottabad aggregates.

**Figure 6:**
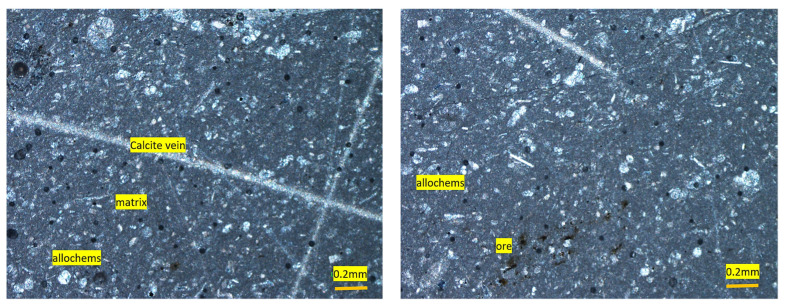
Photomicrographs of wackstone (Bioclastice limestone) showing allochems (Bioclasts) and lime-mud matrix (Abbottabad aggregate).

The aggregate sample was fine- to medium-grained and dark gray to black in color. Based on petrographic analysis, the sample was classified as wackstone (limestone), with calcite forming more than 90% of the rock by volume (Figure 5). Major components were fossils fragments allochems and carbonate mud (Figure 5). The lime mud portion was fine grained, while the bioclasts/fossil fragments ranged from 0.2 to 1.8 mm. The broken fragments of fossils were recrystallized partially to microspar. Quartz and chert (cryptocrystalline silica) and ores were also found, but these formed a very small portion of the sample. Microscopic calcite veins also crosscut the rock samples (Figure 6). Patches of coarse-grained euhedral crystalline calcite were also found occasionally. Rigorous effervescence with diluted HCl further confirms the abundance of calcite (CaCO_3_).

According to Dunham classification [47], these aggregate samples consisted of more than 90% of calcite (CaCO_3_) mineral, and therefore are suitable for use in concrete work as well as with asphalt provided the other required geo-mechanical properties (loss of abrasion, water absorption, compressive strength and porosity, etc.), as per project specifications, also lie within the permissible ranges. Owing to the heterogeneity in the rock types at the quarry, the material engineer is advised to monitor the supplied aggregate from time to time.

#### 4.2.3. Orakzai Aggregate

**Figure 7:**
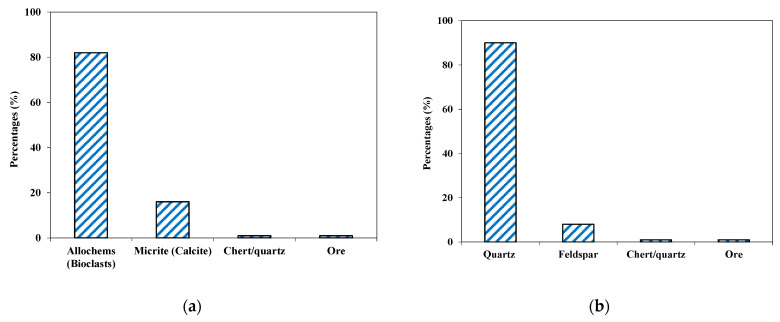
Mineralogical composition of Orakzai aggregate (**a**) limestone, (**b**) quartzite.

This aggregate sample consisted of two groups: limestone and quartzite. Limestone aggregate sample was medium grained and grayish in color. Based on petrographic analysis, the sample was classified as packstone (limestone), with calcite forming more than 90% of the rock by volume (Figure 7a). Major components were fossils fragments (allochems) and lime-mud matrix. The bioclasts/fossil fragments ranged from 0.2 to 1.1 mm, whereas, the lime mud portion was fine grained. The broken fragments of fossils were partially recrystallized to spar. Quartz and chert (cryptocrystalline silica) and ores were also found, but these formed a very small portion of the sample, and hence were in the safe ranges as per ASTM C295 [39].

**Figure 8:**
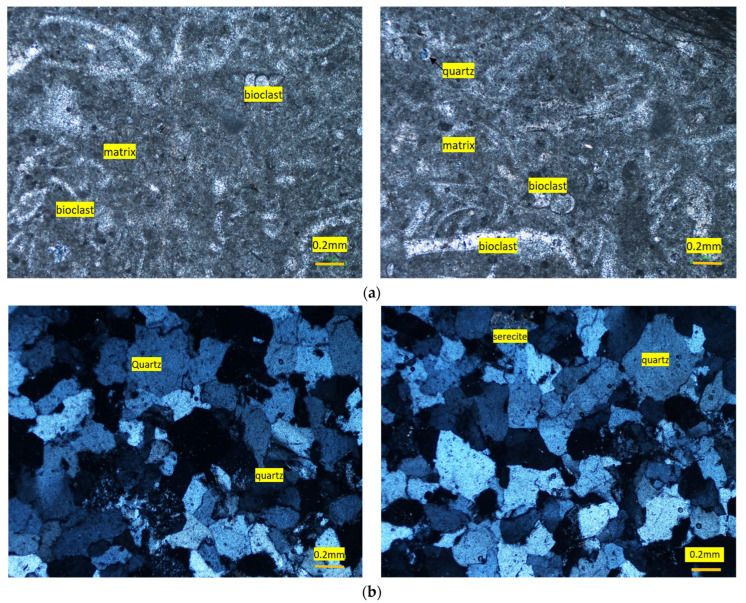
Photomicrographs of Orakzai aggregates (**a**) packstone (bioclastic limestone) showing allochems (Bioclasts) and lime-mud matrix, (**b**) quartzite showing quartz and altered feldspar.

Microscopic calcite veins also crosscut the rock samples (Figure 8). Patches of coarse-grained euhedral crystalline calcite were also found occasionally. Rigorous effervescence with diluted HCl further confirms the abundance of calcite (CaCO_3_).

Quartzite in the tested aggregates was medium grained (0.2 to 0.8 mm) and grayish–whitish in color. Based on petrographic analysis, this sub-group was classified as quartzite, with quartz having 90% of the fragments by volume (Figure 7b). Quartz constitutes the major phase and angular to subrounded, anhedral, inequigranular, and was mostly deformed as demonstrated by their wavy extinction. Feldspar occurred as anhedral, and sub-angular grains which showed alteration to clays and sericite. The broken fragments of fossils were recrystallized partially to microspar. Chert (cryptocrystalline silica) and ores were also found.

Due to the occurrence of strained quartz, these fragments may cause alkali–silica reactivity if used in concrete work. Owing to the heterogeneity in the rock types at the quarry, the material engineer is advised to monitor the supplied aggregate from time to time.

This tested aggregate consisted of about 60% limestone and 40% quartzite minerals. The concentration of deleterious contents for alkali–silica reactivity (ASR) lies in a deleterious category. The aggregate was, therefore, not suitable for use in concrete work with OPC. However, the aggregate may be used in asphalt work if all the other geo-mechanical properties lie within suitable ranges.

#### 4.2.4. Swabi Aggregate

**Figure 9:**
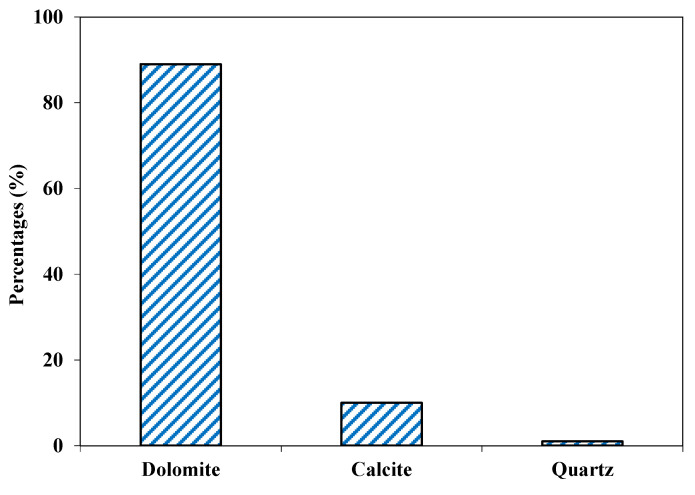
Mineralogical composition of Swabi aggregate.

**Figure 10:**
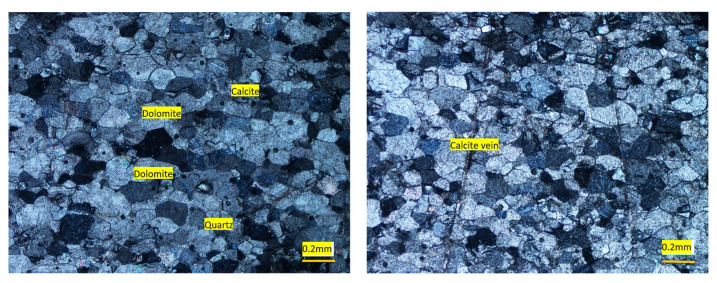
Photomicrographs of calcareous dolostone from Swabi area showing dolomite, calcite, quartz and calcite veins cross cutting the rock.

The analyzed sample dominantly consisted of dolomite (CaMg (CO_3_)_2_) and calcite (CaCO_3_) minerals. The color in dry state was light gray. The concentrations of dolomite and calcite were 89% and 10%, respectively (Figure 9). Texturally, the rock sample consisted mainly of fine- to medium-sized grains in the range of 0.1 to 0.4 mm. There were patches of coarse-grained recrystallized carbonate minerals, mainly calcite. Dolomite was the most abundant mineral phase, showing a subhedral to euhedral shape. Dolomite also occurred as rhomb-shaped crystals. The calcite occurred as medium-sized subhedral grains and constituted the second most abundant mineral. The grain boundaries of calcite interpenetrated with those of other minerals (mainly dolomite) in the thin sections. Quartz was the minor mineral phase and occurred as fine monocrystalline grains which had a subhedral, sub-angular and subspherical shape. Calcite vein, traversing the main matrix, were also occasionally observed (Figure 10).

On the basis of petrography, it was classified as calcareous dolomite. The aggregate can be used with asphalt if the rest of the required standards fall within suitable ranges. The higher amount of dolomite makes this group unsuitable for use in concrete work, as it will cause alkali–carbonate reactivity (ACR) if used with ordinary Portland cement. However, its total percentage was low in the whole aggregate that occurs within the permissible limit for ACR.

#### 4.2.5. Sada Aggregate

**Figure 11:**
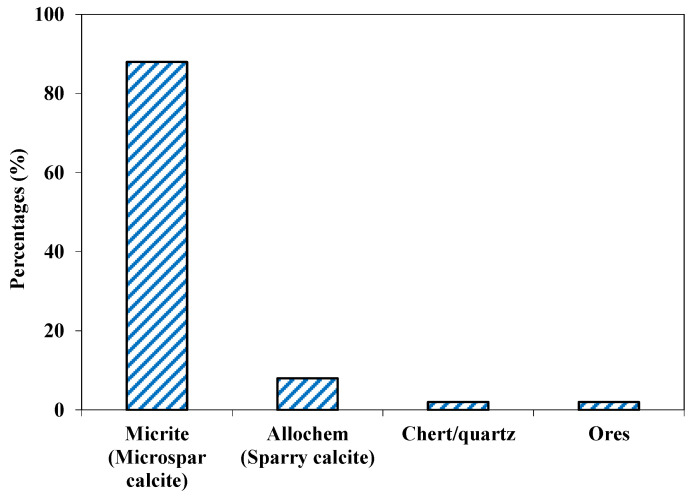
Mineralogical composition of Sada aggregate.

**Figure 12:**
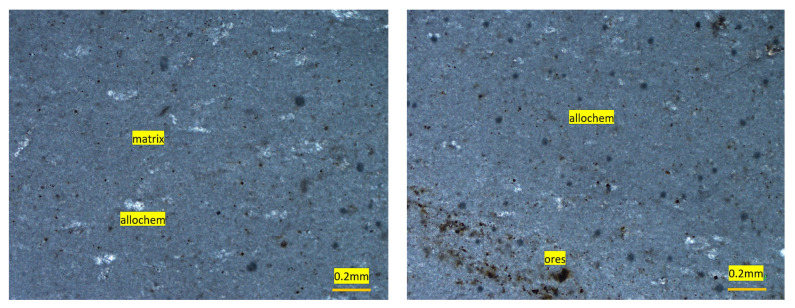
Photomicrographs of mudstone showing few allochems and lime-mud matrix.

This aggregate was fine- to medium-grained (0.1 to 0.4 mm) and showed a brownish gray color. Based on petrographic analysis, these fragments were classified as fine-grained limestone. It can be also classified as mudstone as per Dunham classification [47], where small calcite grains (CaCO_3_) formed about 88% of the sample by volume (Figure 11). A major component was the sparry/micro-sparry calcite (CaCO_3_) formed by neomorphism of fine matrix. Quartz/chert also occurred as subhedral to anhedral grains and they formed a very minor portion (2%) of the sample. Dark color ores, probably iron ores, were also observed (Figure 12).

The ASR potential of aggregates is linked with its mineralogical composition, structural type and nature of silica contents and exposure conditions. The constituents that are susceptible to ASR include the opal (0.50%), cristobalite (1%), chert (3%), strained quartz (5%), volcanic glasses (3%) among others [48]. Conversely, aggregates containing dolomite and limestone do not have reactive components and do not contribute towards ASR [49].

### 4.3. Accelerated Mortar Bar Expansion Results

**Figure 13:**
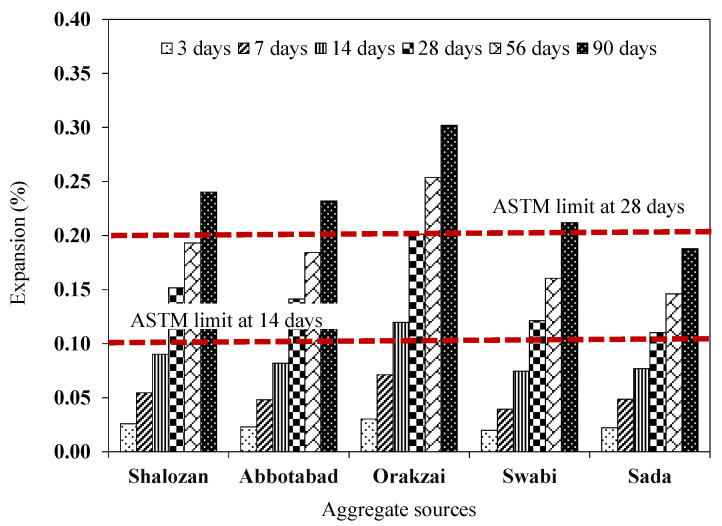
Expansion results of various tested aggregates.

The values of expansion measured from mortar bars were shown in Figure 13. Each result was the average of five identical specimens with a coefficient of variation less than 1.6%, which was within ASTM C1260 [8] limitations.

As the length of the mortar bars began to expand with the increase in the testing period, each source produced a different result, which depends on the existence of reactive minerals. For instance, the Orakzai source had the maximum expansion of 0.302% at 90 days among all the tested aggregate sources. This was mainly because of reactive minerals that were confirmed in the chemical analysis, as well as in the petrographic examination of the aggregates. On the other hand, the Sada source had the minimum expansion of 0.187% at 90 days, indicating its non-reactive nature. In the same way, all other aggregate sources, i.e., Shalozan, Abbottabad, and Swabi showed expansion of 0.240%, 0.232% and 0.211%, respectively, after 90 days of their exposure in ASR conditions.

It was observed that specimens made with Orakzai aggregates showed expansion of 0.119% at 14 days and 0.201% at 28 days. Similarly, expansion results of specimens incorporating Shalozan, Abbottabad, Swabi, and Sada sources were 0.090%, 0.082%, 0.074% and 0.0769% at 14 days, respectively, and 0.152%, 0.141%, 0.121% and 0.110% at 28 days, respectively (Figure 13).

**Figure 14:**
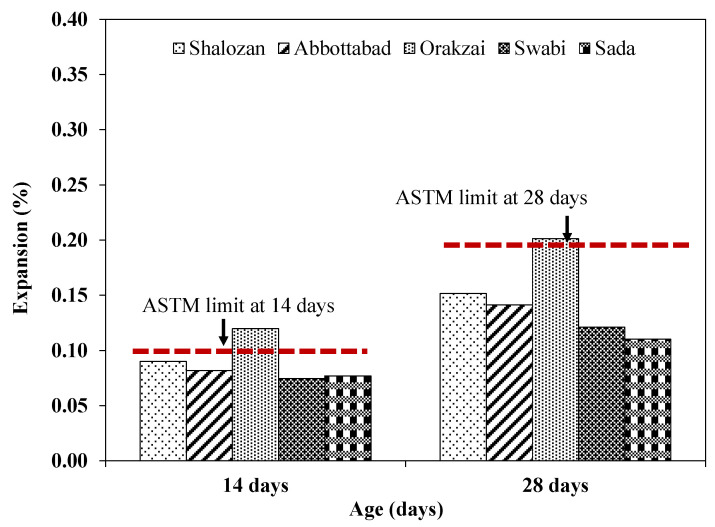
ASTM C1260 limits for the tested aggregates.

Sources were classified as alkali–silica reactive if they expanded more than 0.10% after 14 days and 0.20% after 28 days, according to ASTM C 1260 [8]. As a result of current study findings, it was found that one of these sources (Orakzai) was alkali–silica reactive and unsafe to use, as specified by ASTM C 1260 [8] (Figure 14). Results of Shalozan, Abbottabad, Swabi, and Sada sources confirm that these were safe against ASR. According to previous studies, ASTM C 1260 [8] was proven to be more effective with slow-reacting minerals [7].

Each specimen was visually monitored after the performance of test to investigate the surface cracking and associated damages. It was observed that specimens cast with Orakzai aggregates showed surface cracking after 90 days exposure to ASR conditions. However, no cracking and surface distress was noted for specimens cast with Shalozan, Abbottabad, Swabi and Sada sources aggregates.

**Figure 15:**
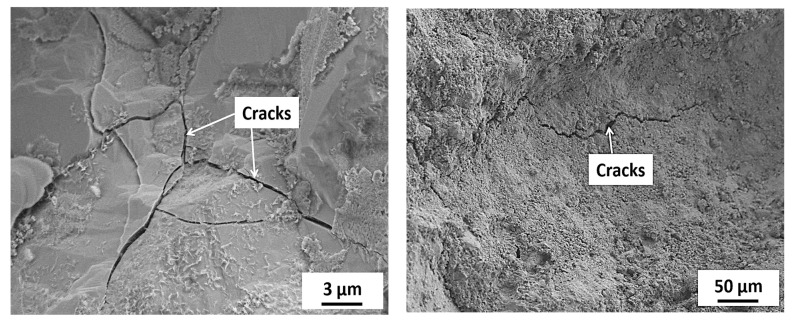
SEM images of specimens incorporating Orakzai source aggregates.

**Figure 16:**
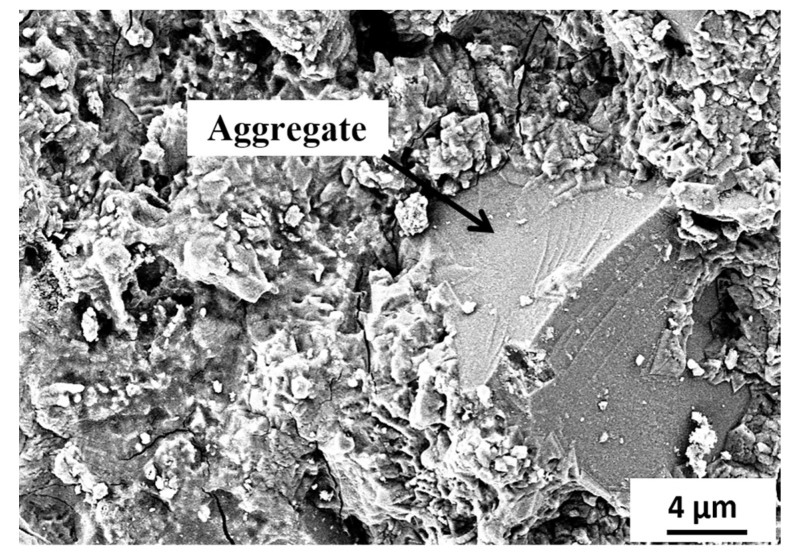
SEM image of specimen around the aggregate particle.

**Figure 17:**
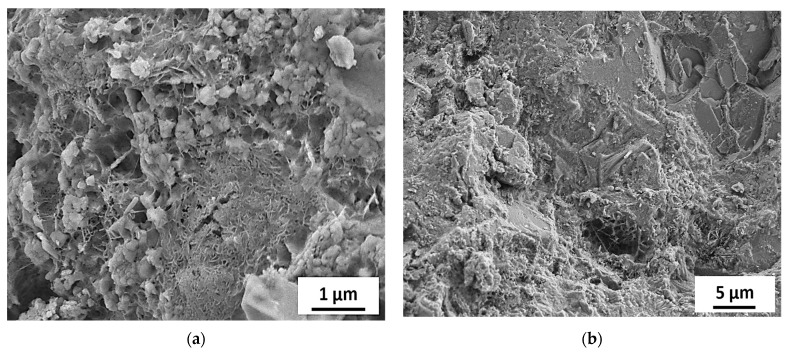
SEM images of specimens incorporating various aggregates (**a**) Abbotabad source aggregates (**b**) Swabi source aggregate.

**Figure 18:**
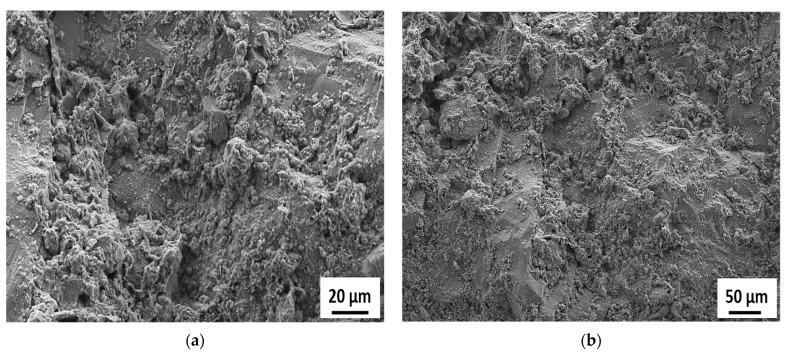
SEM images of specimens incorporating Shalozan aggregates (**a**) 20 µm (Mag. = 500×) (**b**) 50 µm (Mag. = 180×).

**Figure 19:**
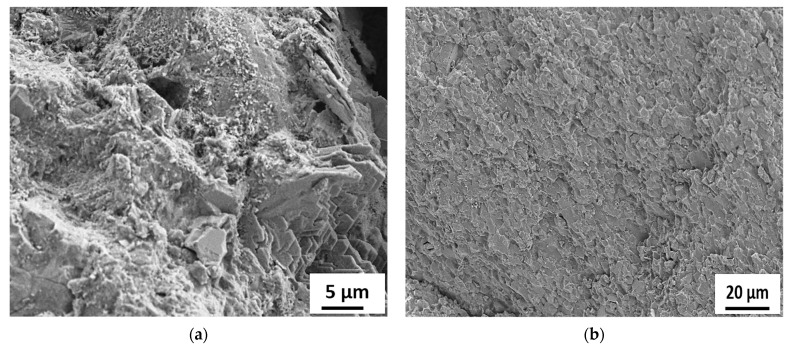
SEM images of specimens incorporating Sada aggregates (**a**) 5 µm (Mag. = 1.92K×) (**b**) 5 µm (Mag. = 500×).

Figure 15, Figure 16, Figure 17, Figure 18 and Figure 19 show the SEM images conducted on the tested specimens. It was observed that specimens incorporating Orakzai aggregates showed microcracking (Figure 15) after exposure to ASR conditions. Figure 16 shows the SEM image around the aggregate particle. Specimens incorporating Shalozan, Abbottabad, Swabi and Sada sources aggregates showed no microcracking cracking due to ASR.

**Figure 20:**
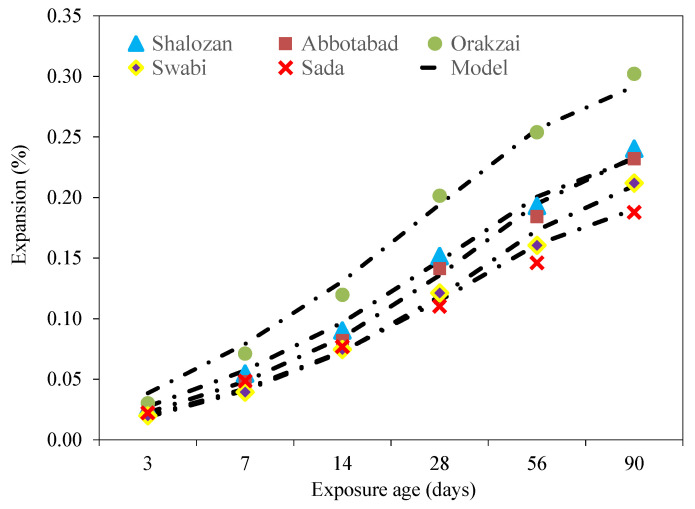
Experimental results versus model prediction of expansion along with exposure ages.

The measured average ASR expansion versus time for the tested aggregates can be mathematically fitted using the following model (Equation (5)) proposed by Islam, 2010 [45].

(5) $$y=\frac{t}{at+b}$$

where *y* is the average ASR expansion, *t* is the time at which expansion was measured and *a* and *b* are the regression parameters. Table 7 shows the regression parameters (*a* and *b*) for the tested aggregates. Figure 20 shows the model prediction (Equation (5)) expansion results versus the experimental values, indicating a satisfactory correlation.

### 4.4. Effect of ASR on Compressive Strength

**Figure 21:**
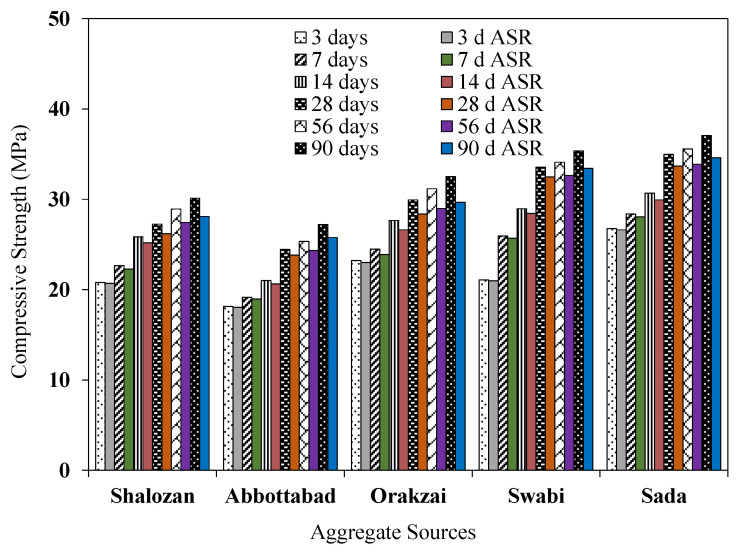
Effect of ASR conditions on compressive strength.

Figure 21 shows the variation in compressive strength of control specimens and specimens under ASR conditions. Compressive strength results were an average of five specimens with a coefficient of variation (COV) of less than 1.86%. Results revealed that, at 28 days, the control specimens (cured under water curing) of Sada source had a maximum compressive strength of 34.98 MPa, whereas Abbottabad source had a minimum compressive strength of 24.46 MPa. At 90 days, the maximum compressive strength of control specimens for Sada source was 37.07 MPa, whereas the Abbottabad source had minimum compressive strength of 27.23 MPa.

The compressive strength of specimens under ASR conditions showed a clear reduction in strength with the passage of time compared to control specimens cured in normal water. For example, the compressive strength of Orakzai aggregate specimens exposed to ASR conditions was reduced by 5.24% at 28 days compared to control specimens. Similarly, the compressive strength of the Abbottabad source decreased by 3.31% at 28 days for specimens exposed to ASR conditions compared to that of the identical specimens placed in water curing. With the passage of time, the compressive strength reduction increases for ASR exposed specimens. For instance, Swabi source specimens have reduction in compressive strength of 3.18% at 28 days and 8.82% at 90 days.

The Orakzai aggregate specimens exhibited the highest reduction in compressive strength under ASR conditions at 90 days, as they contained highly reactive particles observed through petrographic examination and confirmed by expansion measurements as well. The lowest reduction in compressive strength of 5.2% was recorded for specimens made with Sada aggregates at 90 days (Figure 21). A similar reduction in compressive strength was also reported in previous studies. For example, Munir et al. [7] reported compressive strength loss of 22% for Mach hills aggregates under ASR exposure conditions, whereas Tuguwali aggregate reported 16% loss under same circumstances. Marzouk et al. [9] reported overall reduction of 24% in compressive strength of normal-strength concrete at 84 days for the specimens exposed to NaOH solution. Based on this study, it was confirmed that the alkali–silica reaction reduces the compressive strength. The more reactive the aggregate source, the more compressive strength reduces.

### 4.5. Effect of ASR on Modulus of Rupture

**Figure 22:**
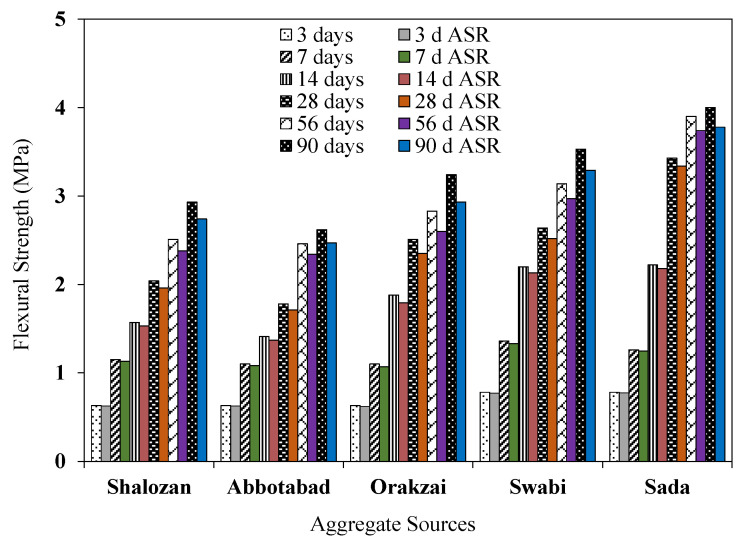
Variation in flexural strength of control samples and the samples under ASR conditions.

Figure 22 shows the difference in flexural strength of control specimens and specimens under ASR conditions. Flexural strength data showed COV less than 2.08%. Due to the ongoing hydration process the flexural strength increased with longer curing period, as expected. Test results revealed that the loss in flexural strength of prism samples under ASR circumstances compared to control samples under water curing. For example, flexural strength of Orakzai aggregate specimens showed 2.51 MPa and 2.35 MPa at 28 days when exposed to control and ASR conditions, respectively. Similarly, the flexural strength of the Abbottabad source decreased by 3.93% at 28 days. The flexural strength decreased further with increase in time. For instance, Swabi source specimens showed a 4.55% decrease in flexural strength at 28 days and 6.80% at 90 days compared to identical specimens placed in water curing. The Orakzai aggregates exhibited a significant drop of 9.5% in flexural strength under ASR conditions at 90 days. However, the flexural strength of Sada samples showed the lowest drop of 5.5% in flexural strength at 90 days. Other tested aggregates sources, i.e., Shalozan, Abbottabad and Swabi, also showed a decrease in flexural strength (6.5%, 5.7% and 6.8% at 90 days, respectively) under ASR conditions. Previous studies also reported a reduction in flexural strength due to ASR exposure. For example, Munir et al. [7] reported an overall loss in flexure strength of the samples tested under ASR exposure in range from 22 to 34%. Marzouk et al. [9] reported loss of flexural strength of up to 24% associated with moderately reactive aggregates. In this study, it was reaffirmed that the alkali–silica reaction reduces the modulus of rupture. The more reactive the aggregate source, the more reduction in the rupture modulus will be observed.

## 5. Conclusions

This study investigates the potential of locally available unexplored aggregates with regard to ASR as per ASTM C1260. The studied aggregates were from Shalozan, Abbotabad, Orakzai, Swabi and Sada. These aggregates were directly procured from their crusher sites. Petrographic analyses were conducted on these aggregates, along with determination of their physical and chemical properties. The expansion of mortar bars incorporating such aggregates caused by ASR was investigated, and the subsequent reduction in compressive and flexural strength on cube and prism specimens under ASR exposure was also examined. Following conclusions are drawn from experimental results:

1. The physical properties of the used cement were within the ASTM and European (EN) criteria. The fineness and surface area of used cement were 96.4% and 2867 cm^2^/gm, respectively, which was greater than 90% and 2250 cm^2^/gm (within ASTM limits). The autoclave expansion of cement was considerably lower than 0.8 percent, according to ASTM C151. Moreover, the physical properties of aggregates sources were within the specified ranges of ATSM standards. Swabi aggregates demonstrated the highest resistance to crushing, abrasion and impact, while Abbottabad aggregates had the lowest impact value. Sada aggregates, on the other hand, showed significant resistance to crushing and abrasion.
2. The presence of all chemical constituents was reported to be within the limits specified in ASTM C114. Orakzai aggregates and Shalozan aggregates have greater silica contents of about 64.5% and 37%, respectively, whereas Abbottabad source has a lower silica content of around 1.55%. Petrographic examination of local aggregates confirmed the presence of reactive minerals—as much as 40% in case of Orakzai aggregate—while other tested sources have minerals in the normal range.
3. Expansion results showed that the specimens made with Orakzai aggregate source exhibited expansion of 0.119% and 0.201% at 14 and 28 days, respectively, indicating its reactive nature as per ASTM C1260, while other tested sources showed less than 0.20% expansion at 28 days. Maximum expansion of 0.302% was observed for specimens incorporating Orakzai aggregate source at 90 days.
4. A decrease in compressive and flexural strengths under ASR exposure was observed. For example, at 90 days, specimens with Sada source aggregates showed a reduction of around 5% in compressive strength under ASR conditions compared to that of the identical specimen placed in water curing. A maximum decrease in flexural strength under ASR exposure of around 9% was observed for specimens made with Orakzai aggregate source at 90 days.

This research will facilitate the construction firms in projects related to concrete infrastructure made up of these local aggregates. Those local quarries of aggregates which are prone to ASR must be avoided in concrete structures or suitable precautionary measures need to be taken before their use. For construction purposes, aggregates from Orakzai, Shalozan and Swabi sources can be used after applying precautionary measures to prevent ASR.

## Figures and Tables

**Table 1:** Previous studies conducted on local aggregates.

Sr #	Studied Aggregates/Rock Formation	Test Performed	Results and Findings	References
1	Sakesar limestone, Pail Padhrar, Tobar valley, Dhak pass.	Los Angeles abrasion value, aggregate impact value, aggregate crushing value, soundness test, specific gravity, unit weight.	It was reported that the Dhak pass and Pail Padhar sources exhibited lowest abrasion values. Due to higher crushing value and soundness test value, Tobar valley and Pail Padhar sources were recommended for surface course in pavements. Water absorption, specific gravity and unit weight of all aggregates were satisfactory.	Hassan et al. [14]
2	Chakdara quarry, Katkala quarry, Gulabad Khwar, Pajkor River at Rani.	Specific gravity, water absorption, bulk density, flakiness index, elongation index, soundness, crushing value, impact value, Los Angeles abrasion and concrete cylinder strength.	The specific gravity and bulk density of the four quarried ranged from 2.67 to 2.72 and 99 lb/ft^3^ to 101 lb/ft^3^, respectively, while the water absorption ranged from 1.58% to 1.92%. The flakiness index and elongation index ranged from 21.1% to 25.1% and 14.7% to 24.2%, respectively. The soundness test values ranged from 3.35% to 5.04%. The aggregate crushing value and aggregate impact value tests showed that aggregates from Gulabad quarry were better, with lowest values of 13.23% and 16.56%, respectively. The concrete cylinder strength of Gulabad aggregate was also highest (2376 psi) among the tested sources.	Hakim et al. [15]
3	Loni Kot area (Karachi-Hyderabad Motorway).	Particle density, water absorption, bulk density, soundness, organic impurities, clay lumps and friable particles, flakiness and elongation index, Los Angeles abrasion test, crushing value and impact value.	It was reported that 77% of fine particles were free from organic impurities. The aggregates soundness value was also within limits specified. The water absorption of fine aggregates was between 0.40 to 2.20% while, that of coarse aggregates was between 0.20 to 0.60%. The specific gravity of fine and coarse aggregates was between 2.42 and 2.72 and 1.95 and 2.87, respectively. The flaky and elongated particles in coarse aggregates were 25% and 27%, respectively. The aggregate crushing value and impact value were reported to be 25% and 28%, respectively. It was reported that the tested aggregates were sound and possessed good resistance against fragmentation and crushing.	Pathan et al. [16]
4	Sheikh hills, Tuguwali hills and Mach hills from the Sargodha region, the Jhelum River at the Lehri Mangla and the Kamser Mountains from Muzaffarabad in Kashmir.	Petrographic analysis, expansion test.	The ASTM C227 was followed to determine mortar bar expansion. The values for aggregates from Sargodha region ranged from 0.05 to 0.07%. However, for aggregates from Jhelum and Kamser sources, the expansion values were less than 0.04%. Using the ASTM C1260, all tested sources of aggregates from the Sargodha region were found to be reactive, with expansion > 0.20%. Petrographic examination also confirmed the reactivity of Sargodha aggregates.	Munir et al. [17]
5	Margalla crush, Sargodha crush, Mangla crush and Barnalla crush.	Specific gravity, water absorption, bulk density, crushing value, impact value, concrete cylinder strength, splitting tensile strength and flexural strength.	The water absorption of Sargodha and Barnala crush was less than the Margalla and Mangla crush, while the specific gravity of Margalla crush was the highest. However, the bulk density of Margalla crush was the lowest of them all. Sargodha crush exhibited least values of impact value (11.6%) and crushing value (17.9%). Out of all four, Margalla crush exhibited the highest compressive strength (26.3 MPa) and flexural strength (4.90 MPa). The splitting tensile strength of Barnala crush was found to be highest.	Munir et al. [18]
6	Malikhore formation (Lasbela and Khuzdar districts).	Bulk density, water absorption, Los Angeles abrasion, compressive strength, alkali–silica reactivity, flakiness and elongation index and petrographic analysis.	The specific gravity and water absorption were found to be 2.74 and 0.28%, respectively. The Los Angeles abrasion test value was 23% and compressive strength was 6179 psi. The petrographic analysis showed that these aggregates might be considered suitable for use in concrete production.	Naseem et al. [19]
7	Obhan Shah quarry (OSQ), Chattan Shah quarry (CSQ), Goal Pahari quarry (GPQ), Darak quarry (DQ) and Jara Takar quarry (JTQ).	Specific gravity, bulk density, flakiness and elongation index, water absorption, crushing value, impact value, abrasion value and compressive strength.	The specific gravity values of all sources were within allowable limits; however, the bulk density of all sources was below 2400 kg/m^3^. The flakiness index of all sources was above the limit (15%); however, the elongation index was below the maximum limit of 25%. The water absorption, crushing value, impact value and abrasion value of all sources was within standard limits. CSQ exhibited highest 28-day compressive strength (38.2 MPa), while JTQ exhibited lowest compressive strength (20.8 MPa).	Qureshi et al. [20]
8	Margala hill limestone (MH), Lockhart limestone (LT), Kawagarh (KW), Sammana Suk (SM) and Shekhai (SH).	Impact value, flakiness index, elongation index, Los Angeles abrasion, density, water absorption and petrographic analysis	It was reported that all the physical and mechanical properties were within the limits set by BS and ASTM standards. KW showed the highest value for specific gravity. KW and SM showed the lowest impact value and Los Angeles abrasion values.	Naeem et al. [21]
9	Hajra, Kamser, Arja, Margalla and Sargodha crush.	Specific gravity, water absorption, unit weight, flakiness and elongation index, impact value, crushing value, compressive strength and tensile strength.	The specific gravity of Kashmir sources (Hajra, Kamser and Arja) ranged between the values exhibited by Margalla and Sargodha crush, while the water absorption of Hajra and Arja was the highest and for Kamser, it was the lowest. The flakiness index and elongation index of all Kashmir sources were below the BS limits. The impact value and crushing value of Kashmir sources ranged between 10 and 15% and 15 and 23%, respectively. Kamser aggregates showed the lowest compressive and tensile strength values. Arja aggregates showed the highest compressive and tensile strength values.	Siddiqi et al. [22]
10	Bara River, Basi, LoyeKhawar, Zangali/JaniKhawar.	Bulk density, soundness, Los Angeles abrasion, ASR, petrographic examination.	The bulk density of all four aggregates was within the range from 2.3 to 3.1. The soundness test values for Bara river aggregates, Basi, Zangali and Loye Khawar were 13.05, 6.61, 8.94 and 17.69, respectively. The abrasion values of Bara river aggregates, Basi, Zangali and LoyeKhawar were 21.2, 18.5, 24 and 20, respectively. Samples from all four quarries were found to be innocuous and no expansion was found. Petrographic examination showed no signs of unstable silica and reactive carbonates.	Ayub et al. [23]
11	Jurana formation, Sakesar limestone.	Specific gravity, water absorption, soundness test, Los Angeles abrasion, moisture content.	The specific gravity of aggregates sources ranged between 2.62 and 2.70, while the water absorption was from 0.44% to 1.30%. Soundness test ranged between 2.15 to 8.47% after 5 cycles of immersion. The abrasion value ranged between 18.6% and 29.4%. The maximum dry density ranged from 143 lb/ft^3^ to 144.8 lb/ft^3^. The optimum moisture content was found to be between 5.4% and 5.6%. The tested aggregates were recommended for road construction.	Gondal et al. [24]
12	Allai aggregate	Bulk density, specific gravity, water absorption and ASR.	Although all engineering properties were found to be as per standard limits, the aggregates were found to possess alkali–silica potential. It has been recommended that these aggregates can be used along with low alkali cement, fly ash and slag in concrete or other mitigating strategy.	Ahsan et al. [25]
13	Girdue limestone, Sakhi Sarwar, Pitok quarry, Uzman quarry at Nullah Zungi, Khalgeri Mullah quarry.	Specific gravity, water absorption, soundness test, Los Angeles abrasion, moisture content, CBR value.	The specific gravity of aggregates sources ranged between 2.61 and 2.69, while the water absorption ranged from 0.57% to 1.65%. Soundness test ranged between 1.80 to 3.77%. The abrasion value ranged between 17.9% to 30.6%. The maximum dry density ranged from 143.7 lb/ft^3^ to 144.9 lb/ft^3^. The optimum moisture content was found to be between 5.2% and 5.4%. California bearing ratio was found to be between 84.4% to 99.2%. The sources from Girdu formation were reported to be excellent for surface treatment and concrete work.	Gondal et al. [26]
14	Chiniot, Margala, Sikhanwali, Takial and Khairabad.	Crushing value, abrasion value, specific gravity, porosity and particle shape index.	The crushing value of all samples ranged between 21.78 to 29.20%. Los Angeles abrasion value ranged between 16.3 and 25.46%. The impact value ranged between 12.73 and 18.65%. Margala and Chiniott sources exhibited the lowest values for porosity, crushing value and impact value tests and the highest values for specific gravity. However, the percentage of flaky and elongated particles was the highest in case of Margalla source aggregate.	Kamal et al. [27]

# representing the No. (Serial No.).

**Table 2:** Location of aggregate sources.

Sr. No.	Aggregate Source	Latitude	Longitude
1	Shalozan Parachinar	33°56′7″ N	70°1′15″ E
2	Sada Lower Kurram	33°40′2″ N	70°19′28″ E
3	Orakzai Agency	33°42′26″ N	70°50′1″ E
4	Abbottabad Aggregate	34°08′49″ N	73°12′52″ E
5	Swabi Aggregate	34°06′60″ N	72°27′60″ E

**Table 3:** Aggregate samples.

Aggregate Sources	Crusher Site	Aggregate Sample
Shalozan Parachinar	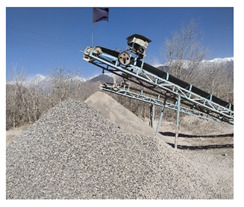	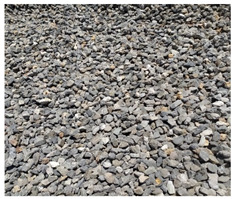
Sada Lower Kurram	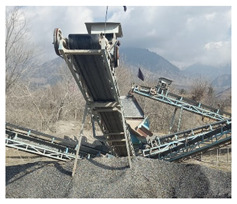	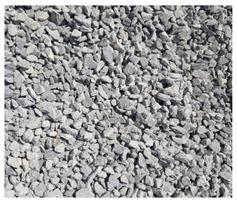
Orakzai Agency	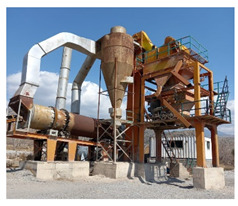	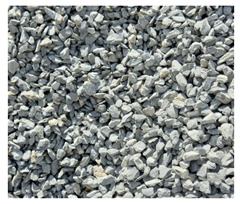
Abbottabad	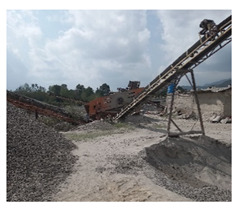	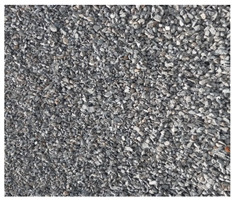
Swabi	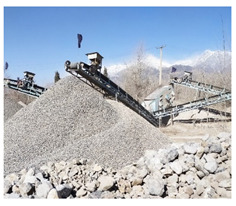	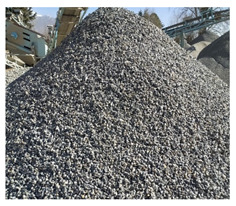

**Table 4:** Physical properties of cement.

Properties	Standards	Values	Limits
Standard consistency	ASTM C187 [28]	22%	-
Initial setting time	ASTM C191 [29]	95 min	Greater than 45 min
Final setting time	ASTM C191 [29]	175 min	Less than 375 min
Fineness (passing No. #200)	ASTM C184 [30]	96.4%	Minimum 90%
Fineness (Blaine air Permeability)	ASTM C204 [31]	2867 cm^2^/g	Minimum 2250 cm^2^/g
Soundness	EN 196-3 [32]	0.7 mm	Maximum 10 mm
Autoclave expansion	ASTM C151 [43]	0.072%	Maximum 0.8%

# representing the No.

**Table 5:** Physical properties of tested aggregates.

Aggregates	Bulk Density (kg/m^3^)	Specific Gravity	Water Absorption	Impact Value	Crushing Value	Abrasion Test
Shalozan	1430	2.62	0.58	25.28	22.14	29.8
Abbottabad	1470	2.63	0.37	26.95	14.07	24.8
Orakzai	1450	2.67	0.35	20.12	13.11	24.8
Swabi	1500	2.81	0.54	18.53	19.22	21.9
Sada	1410	2.70	0.22	23.19	8.15	31.2

**Table 6:** Chemical composition of used aggregates.

Constituents	Shalozan	Abbottabad	Orakzai	Swabi	Sada
CaO (%)	9.4	14.9	7.6	17.02	11.1
MgO (%)	0.2	1.3	1.5	1.1	1.8
SiO_2_ (%)	37	1.55	64.50	3.20	1.64
SO_3_ (%)	0.29	0.16	0.41	0.20	0.23
Al_2_O_3_ (%)	1.60	1.40	0.85	5.65	0.52
Fe_2_O_3_ (%)	0.50	0.74	0.25	2.34	0.28
L.O.I (%)	27	42.62	14.82	44.34	36.05

% mentioned is the weight percentage.

**Table 7:** Regression parameters for the tested aggregates.

Aggregates	*a*	*b*
Shalozan Parachinar	3.20	100
Sada Lower Kurram	2.90	125
Orakzai Agency	2.65	70
Abbottabad Aggregate	3.10	150
Swabi Aggregate	3.70	140

## Data Availability

Not applicable.
